# A proposed research agenda for the study of educational inequities in diet quality

**DOI:** 10.1017/S1368980025101663

**Published:** 2026-01-14

**Authors:** Dana Lee Olstad, Lynn McIntyre

**Affiliations:** Department of Community Health Sciences, Cumming School of Medicine, University of Calgary, Calgary, AB T2N 4Z6, Canada

**Keywords:** Educational attainment, Diet quality, Policy, Equity, Research agenda

## Abstract

Educational attainment is a key determinant of diet quality. The overarching pathways (i.e. theories and mechanisms) through which educational attainment shapes diet quality remain largely unexplored in the nutrition literature, and the most salient pathways likely differ across time, populations and socio-economic and political contexts. This commentary proposes a research agenda and outlines methodological considerations that are intended to better illuminate the educational attainment–diet quality relationship. From an extensive review of the literature, which led to two publications pertinent to the topic, we identified three major research gaps that should be addressed to better understand how educational attainment stratifies diet quality to guide interventions and inform equity-enhancing policies: (1) interrogating the construct of educational attainment; (2) comparative population–level and subgroup studies; and (3) root cause analyses and structural reforms. We also discuss methodological considerations needed to inform future studies of associations between educational attainment and diet quality.

The triple jump in problem-based learning is a type of assessment that evaluates the ability to organise information, formulate hypotheses and identify learning issues that can help to reformulate a problem^(1)^. We recently published two papers that contribute to the first two steps of the triple jump in furthering understanding of dietary inequities through the lens of educational attainment^(2,3)^. The purpose of this commentary is to contribute to the last step by proposing a research agenda and outlining methodological considerations based on what needs to be learned about the educational attainment–diet quality relationship to guide interventions and subsequently inform equity-enhancing policies.

Our first paper showed that educational attainment is a key determinant of diet quality^(2)^. While diet quality is patterned by household income in high-income nations, educational attainment appears to be at least as important – if not more important – in structuring dietary inequities. A key learning from this paper is that economic factors play a more modest role in stratifying dietary inequities than commonly cited, indicating that the mechanisms that explain dietary inequities extend beyond the material disadvantages imposed by inadequate incomes. As such, equity-oriented nutrition research needs to move away from its current focus on income.

Our second paper described overarching pathways through which educational attainment may act as a determinant of diet quality^(3)^. These pathways comprised seven theoretical perspectives (i.e. fundamental cause theory, capital theory, signalling theory, commodity theory, resource substitution theory, resource multiplication theory and the psychosocial hypothesis) and five categories of mediating mechanisms (i.e. individual knowledge, cognitive and non-cognitive skills and traits and sense of personal control; psychosocial factors; economic and employment resources; socio-economic, political and historical contexts; and social distinction). The evidence reviewed in this paper suggests that educational inequities in diet quality are not simply the consequence of the more highly developed knowledge, skills and cognitions associated with higher educational attainment. Rather, multiple theories and mechanisms likely explain educational inequities in diet quality and the most salient overarching pathways likely differ across time, populations and socio-economic and political contexts. To date, these theories and mechanisms remain largely unexplored in the nutrition literature. In what follows, we reflect on the implications of our findings and propose a research agenda, inclusive of methodological considerations, that seeks to advance understanding of the education–diet quality relationship.

## Research priorities

During our extensive review of the literature for the two papers that underpin this commentary^(2,3)^, we identified three major research gaps that should be addressed to better understand how educational attainment stratifies diet quality. First, we encountered empirical and conceptual questions related to what educational attainment purports to measure – an overarching gap that must be filled within the broader health equity literature. Second, we identified the importance of conducting comparative studies of education–diet associations both between and within nations to elucidate key drivers of dietary inequities across time and place but noted that such studies remain relatively few. Finally, we encountered a largely pragmatic approach whereby studies investigated the impacts of downstream programmes to mitigate the adverse dietary effects of lower educational attainment, while neglecting to study the structural forces that make higher education elusive for disadvantaged populations.

1. Interrogating the construct of educational attainment

Just as individuals tend to search for their lost keys under the light post, nutritional epidemiologists often search for determinants of dietary inequities using socio-economic indicators that are the most readily available and quantifiable. Thus, traditional measures of education focus on attainment and years of schooling, which are convenient to measure but fail to capture other aspects of education such as the type, timing and quality of education received^(4)^. Zajacova and Lawrence^(5)^ suggest that research should devote greater attention to understanding whether and how other aspects of education beyond attainment of a credential shape health-related outcomes. For instance, do additional years of schooling that do not result in an additional credential influence diet quality? What role, if any, do institutional type, the quality and content of instruction received and the social environment of the classroom play in relation to dietary inequities? How do the social networks that more highly educated adults develop while in school shape their dietary patterns^(6)^? In considering these questions, studies might be better positioned to uncover mechanisms of educational differentiation^(7)^ that shape diet quality.

Traditional measures of educational attainment also assume that each year of education or credential has the same worth^(4)^. However, a year of education at 6 years of age does not offer the same benefit as a year of post-secondary education completed at the age of 20^(4)^. A high school diploma earned in 1970 does not confer the same knowledge and skills as one earned in 2020. The quality of education received also differs between specialisations and institutions. Traditional measures of education also relate exclusively to formal education, failing to capture informal education that may be acquired in workplaces and other venues. In an era of educational expansion (i.e. the significant increase in the proportion of the population with advanced education that occurred between the 1940s and 1970s)^(8,9)^ where post-secondary education has become normative, it is likely that dietary inequities will emerge according to broader dimensions of the educational experience. Thus, research will need to encompass these harder-to-quantify but equally relevant indicators of the educational experience.

As explained in our second paper^(3)^, current assessment of education–diet quality associations is based on the notion that diet quality improves with attainment of specific diplomas/degrees (i.e. signalling theory)^(10,11)^. By contrast, human capital theory posits continuous improvements in diet quality with each additional year of education^(12)^. Studies that investigate the functional form of educational gradients in diet quality will be essential to adjudicate between the human capital and signalling perspectives^(6)^. For instance, evidence that diet quality improves linearly with additional years of schooling supports a human capital perspective that education imparts valuable knowledge, skills and access to resources that improve diet quality regardless of whether a specific credential is conferred. Evidence that diet quality improves at particular inflection points – such as at 12 and 16 years – supports the signalling perspective that attainment of a high school diploma and a bachelor’s degree are what matters for health. Evidence that diet quality improves at these inflection points, yet also improves linearly between these points, would support both theories. Longitudinal studies would be helpful to answer these questions.

1. Comparative population-level and subgroup studies

Although Western nations share many similarities in terms of educational attainment, economic development, systems of governance and civic institutions, they also differ in ways that are decisive for educational gradients in diet quality, such as the timing and extent of educational expansion, the structure of their educational systems (e.g. tied to employers *v*. independent), social support policies and patterns of social mobility. Future studies should continue to follow trends in educational inequities in diet quality in a variety of nations, including at subnational levels, to enable comparisons of how dietary inequities emerge at different times and places to identify how aspects of socio-economic and political contexts such as these influence the size and strength of education–diet associations. Such studies can generate hypotheses for further empirical testing of specific policies that may have contributed to these variations.

Within countries, less educated groups are not homogeneous. It is important to understand how educational attainment interacts with age, gender, ethnicity, employment status, immigration status and other factors to shape diet quality, consistent with a precision public health approach^(7)^. It is also important to examine whether the mechanisms that explain educational gradients differ among these subgroups. For instance, income may explain a greater proportion of educational gradients in diet quality for those who are less socially integrated.

Finally, we encourage investigators to move away from deficit-oriented thinking and towards strengths-based approaches. Thus, rather than investigating factors that impoverish the lives and diet quality of less educated adults, studies could examine how higher education increases access to health-enhancing resources that promote healthy dietary patterns. Resiliency factors in subgroups attaining higher diet quality despite disadvantage should be sought in a way that recognises that people who do so are overcoming structural constraints in exercising positive agency over their circumstances^(13)^.

1. Root cause analyses and structural reforms

Public funding is limited, and thus it is essential to ensure that the most important mechanisms are targeted in policies that seek to reduce educational inequities in diet quality. In order to do so, studies should systematically examine the plausibility of each of the mediating mechanisms we posited and their joint contributions to dietary inequities, with due consideration to historical, sociodemographic, socio-economic and political contexts. If such studies are to better inform population-based interventions, they should also be conducted and interpreted in a way that avoids offering individual-level solutions to what are fundamentally structural problems.

Given that the determinants of educational attainment at a population level are not the same as the determinants of inequities in educational attainment, studies of broader upstream policies that promote attainment of higher education at a population level, along with studies of policies that address the factors that govern the unequal distribution of educational attainment, will be needed to understand optimal points of intervention. This corresponds to the distinction Graham makes between the social determinants of health and the social determinants of health inequities^(14)^. Social policies that improve educational attainment at a population level (e.g. by reducing tuition fees; ensuring access to high-quality early childhood education) are essential but may not reduce dietary inequities if they do not have greater effects among the least educated. Ultimately, remedying educational inequities that drive dietary inequities will require upstream structural reforms to the way in which power, influence, income and other social determinants of health are allocated in society. Investigating the impact of such policies is a complex endeavour that will require multidisciplinary perspectives.

## Methodological considerations

Finally, several methodological considerations arise in the future study of associations between educational attainment and diet quality.

- Subjective and other indicators of educational experiences beyond attainment of a credential should be used to discern broader mechanisms underlying educational inequities in diet quality.
- Studies should go beyond simple associations to examine which policies reduce educational inequities in diet quality by using, for example, difference-in-differences approaches.
- Multivariate analyses should compare the strength of associations between income and diet quality and education and diet quality with and without mutual adjustment to understand the extent to which income may drive educational inequities in diet quality.
- Mediation analyses should examine factors that mediate associations between educational experiences and diet quality to identify more proximal points of intervention that could reduce dietary inequities in the short term.
- Longitudinal studies should investigate how policy interventions that affect educational attainment influence diet quality over the life course.
- Intersectional approaches should be undertaken to understand how educational attainment intersects with other aspects of disadvantage.

### Conclusion

Educational attainment, diet quality and dietary inequities are the net outcomes of policy choices. This commentary articulates a research agenda to understand and address the broader context in which dietary inequities emerge and are reproduced over time.
